# Dinutuximab beta combined with chemotherapy in patients with relapsed or refractory neuroblastoma

**DOI:** 10.3389/fonc.2023.1082771

**Published:** 2023-02-03

**Authors:** Aleksandra Wieczorek, Anna Zaniewska-Tekieli, Karoline Ehlert, Katarzyna Pawinska-Wasikowska, Walentyna Balwierz, Holger Lode

**Affiliations:** ^1^ Pediatric Oncology and Hematology, Jagiellonian University Medical College, Krakow, Poland; ^2^ Pediatric Oncology and Hematology, University Children's Hospital of Krakow, Krakow, Poland; ^3^ Pediatric Hematology and Oncology, University Medicine Greifswald, Greifswald, Germany

**Keywords:** neuroblastoma, relapsed, refractory, dinutuximab beta, chemotherapy

## Abstract

Prognosis in children with refractory and relapsed high-risk neuroblastoma is poor. Only a minority of patients obtain remission when treated with second-line chemotherapy regimens. Chemotherapy combined with anti-GD2 antibodies has previously been shown to increase response and survival rates. We retrospectively analyzed a cohort of 25 patients with relapsed or refractory high-risk neuroblastoma who were treated with irinotecan/temozolomide chemotherapy in combination with the anti-GD2 antibody dinutuximab beta. The therapy resulted in an objective response rate of 64%, with 32% of patients achieving a complete response. Response to treatment was observed in patients with refractory disease (n=5) and those with first (n=12) or consecutive (n=8) relapses, including patients with progressing disease. In four patients, best response was achieved after more than 5 cycles, suggesting that some patients may benefit from prolonged chemotherapy and dinutuximab beta treatment. Fourteen of our 25 patients had previously received dinutuximab beta, four of whom achieved complete response and six partial response (objective response rate 71%). The therapy was well tolerated, even in heavily pre-treated patients and those who had previously received dinutuximab beta treatment. Toxicities were comparable to those previously reported for the individual therapies, and no discontinuations due to toxicities occurred. Combination of chemotherapy with dinutuximab beta is a promising treatment option for patients with relapsed or refractory high-risk neuroblastoma and should be further explored in clinical studies.

## Introduction

Neuroblastoma is a malignancy of the sympathetic nervous system that commonly affects children under the age of five years (1). Approximately half of the patients with neuroblastoma are diagnosed with a clinically aggressive, high-risk form of disease (2). Despite advances in the first-line treatment of high-risk neuroblastoma, 10–20% of patients do not respond to treatment, and 50–60% relapse (3). Relapsed/refractory neuroblastoma has a poor prognosis and was previously considered fatal (3). In a meta-analysis of data from Phase II trials, median overall survival (OS) was 27.9 months for patients with refractory neuroblastoma and 11.0 months for patients with relapsed neuroblastoma (4). A large International Neuroblastoma Risk Group (INRG) database analysis reported a 5-year OS rate of 20% after the first relapse in patients with relapsed neuroblastoma, which was dependent on the time to relapse and the disease stage at diagnosis (5).

To date, there are no established treatment options for patients with relapsed or refractory neuroblastoma. Initial treatment regimens include chemotherapy combinations distinct from those previously administered, usually based on temozolomide and irinotecan or topotecan (4, 6, 7), or more intensive regimens such as ifosfamide, carboplatin and etoposide (ICE) or topotecan, vincristine, and doxorubicin (TVD) (8, 9). However, many patients do not respond to treatment for relapsed/refractory disease or their disease progresses after an initial response (4). It is also unclear whether consolidation therapy should be given to patients with relapsed or refractory neuroblastoma who achieve remission and, if so, which treatment regimen should be administered (10–12). Treatment with megachemotherapy and autologous stem cell transplantation (ASCT) may be challenging in this population, as most patients are heavily pre-treated and have often undergone ASCT or tandem ASCT, according to a previous experience by the Children’s Oncology Group (COG) (13, 14). In addition, patients with refractory disease or early relapse often experienced toxicities with previous intensive treatment (13, 15, 16).

The combination of immunotherapy and chemotherapy may be a suitable treatment option for patients with relapsed or refractory neuroblastoma. Immunotherapy with the anti-GD_2_ monoclonal antibody dinutuximab beta is the standard of care maintenance treatment in patients with high-risk neuroblastoma in the first-line setting (17–20). Patients with relapsed or refractory high-risk neuroblastoma have also been shown to benefit from dinutuximab beta maintenance therapy (International Society of Paediatric Oncology European Neuroblastoma Group [SIOPEN] long-term infusion study), with improvements observed in both soft tissue lesions and osteomedullary disease (21, 22). However, in both settings, dinutuximab beta was used only in patients with stable disease (17–22). The combination of dinutuximab, an anti-GD_2_ antibody similar to dinutuximab beta, with irinotecan and temozolomide plus granulocyte-macrophage colony-stimulating factor (GM-CSF) was associated with an objective response rate (ORR) of 53.0% in the refractory/relapsed setting in an initial randomized study (COG ANBL1221 trial) and 41.5% in its non-randomized expansion cohort (23, 24). In the BEACON study, a recently completed, randomized Phase II trial investigating dinutuximab beta combined with temozolomide/topotecan versus chemotherapy alone in patients with relapsed/refractory neuroblastoma, resulted in an ORR of 35% for chemoimmunotherapy and 18% for chemotherapy only (25). In addition, promising early response data have been reported for the anti-GD_2_ antibody hu14.18K322A plus GM-CSF as well as dinutuximab plus GM-CSF combined with induction chemotherapy in the first-line setting (26, 27).

We report the use of dinutuximab beta and chemotherapy in the treatment of patients with relapsed or refractory high-risk neuroblastoma as part of two compassionate use programs.

## Materials and methods

### Patients and treatment

We carried out a retrospective review of the clinical charts of patients with relapsed or refractory high-risk neuroblastoma, who received dinutuximab beta immunotherapy combined with chemotherapy as part of compassionate use programs at one of two centers, one in Krakow, Poland, and the other in Greifswald, Germany, between December 2017 and October 2021. Patients were classified as having high-risk neuroblastoma based on the International Neuroblastoma Staging System (INSS) classification system (28, 29), i.e. if they were ≥12 months of age and had INSS stage 4 neuroblastoma, or if they had INSS stage 2, 3, 4 or 4S neuroblastoma with *MYCN* amplification (28, 29). Patients with disseminated relapse, irrespective of age and stage at diagnosis were also included. Patients were also required to have measurable or evaluable disease.

As there are no standard treatment options for patients with relapsed or refractory neuroblastoma, chemoimmunotherapy consisting of dinutuximab beta and chemotherapy was proposed for patients for whom other options were ineffective. Lack of efficacy was defined as either lack of response to therapy or relapse/progression after initial response. Initially, chemoimmunotherapy was only used to treat patients who had previously had no response to treatment of relapsed/refractory disease or had consecutive relapse or progression. Over time, as data indicated that chemoimmunotherapy was effective and well tolerated in this patient population, chemoimmunotherapy was also used to treat patients who were experiencing their first relapse/progression as well as refractory patients.

The treatment of earlier relapses/progression (if any) was not standardized and was based on the standard of care at the institution in which the patient was treated (**Supplementary Table 1**).

Dinutuximab beta was given as continuous long-term infusion of 10 mg/m^2^/day on days 2–6 of each 21-day cycle. Chemotherapy was given on days 1–5 of each cycle. Dinutuximab beta and chemotherapy were administered in parallel on days 2–5: either two-lumen catheters were used and drugs were given *via* two separate lumens or if the child had a port, the peripheral intravenous access was used for irinotecan. Initially, 5 treatment cycles of dinutuximab beta plus chemotherapy were planned, but the definitive number of cycles depended on response to treatment, tolerability and further planned therapy. If well tolerated, at least 2 further treatment cycles were given to patients with complete response (CR) or stable disease (SD). In those who were responding to therapy but had not yet achieved CR, the additional treatment cycles were given until CR, disease progression, SD in two consecutive evaluations, or intolerable toxicity. Intolerable toxicity was generally considered as any grade 3 or 4 toxicity that did not improve to grade 1 or 2 prior to the next treatment cycle, or grade 4 hematologic toxicities that did not improve between treatment cycles. When consolidation treatment was planned, the number of cycles of dinutuximab beta plus chemotherapy was based on treatment response – treatment was complete when the response was sufficient to allow megachemotherapy to be administered, or when disease progression was diagnosed. All patients received the standard supportive treatment recommended when administering dinutuximab beta by long-term infusion (30, 31).

Informed consent from the parents/legal guardians of the patient or the patient themselves was obtained for treatment. The compassionate use program was approved for each patient individually by the Bioethical Committee of the District Medical Chamber in Krakow, Poland or by the local committee in Greifswald, Germany.

### Assessments and outcomes of interest

Tumor response was evaluated at baseline, following 2 and 5 cycles of dinutuximab beta plus chemotherapy, every 2–3 cycles thereafter in patients receiving >5 treatment cycles, and at any time when progression/relapse was suspected, using the International Neuroblastoma Response Criteria (32) for metastatic lesions (32). For the primary tumor, SD was defined as tumors that did not increase or decreased in size by >25%, progressive disease (PD) as tumors that increased in size by >25%, a partial response (PR) was a decrease in tumor size of >25% with tumor(s) remaining, and a CR was the absence of tumors, according to the guidelines in the LINES protocol. The evaluation was done locally during a meeting of oncologists, surgeons and radiologists. Tumors were assessed radiographically in patients with measurable disease using computed tomography and/or magnetic resonance imaging. Patients with iodine-123 or iodine-131-meta-iodobenzylguanidine (MIBG)-positive lesions were evaluated for MIBG response (every 3 cycles after cycle 5). Patients with MIBG non-avid disease were examined with positron emission tomography. Bone marrow involvement was assessed bilaterally using routine cytomorphologic examination, and histopathologic examination with immunostaining.

Progression-free survival (PFS) was defined as the time between the initiation of chemoimmunotherapy with dinutuximab beta plus chemotherapy and the first occurrence of relapse, or disease progression. OS was defined as the time from the initiation of chemoimmunotherapy until death from any cause. Treatment failure was defined as the presence of a new lesion or progression of size or number of known lesions in relapsed patients or as lack of response sufficient for therapy continuation according to HR-NBL SIOPEN protocol in refractory patients.

Patients were monitored for adverse events (AEs) according to the Common Terminology Criteria for Adverse Events (CTCAE) version 4.0. Pain was evaluated using the Wong-Baker FACES Pain Rating Scale, where 0 indicates no pain, and 10 the worst pain imaginable.

### Statistical analysis

The data cut-off was January 31, 2022. Survival curves were estimated using the Kaplan–Meier method and were compared using a log-rank test (*p*<0.05 was considered statistically significant) (33, 34). For the survival analyses, patients were censored at the date of the last assessment. The 1-year and 3-year PFS and OS rates were estimated according to the Kaplan–Meier method (33, 34), and standard errors were calculated according to the Peto method (35). Differences between groups were analyzed using the chi-squared test.

The effect of the following factors on response and survival after chemoimmunotherapy was analyzed using univariate analysis with logistic regression (odds ratio [OR]; *p*<0.05 was considered statistically significant): age <18 months at diagnosis, *MYCN* amplification, time of relapse (during versus after first-line therapy), type of relapse (metastatic versus combined), prior treatment with dinutuximab beta and prior treatment with megachemotherapy plus ASCT.

## Results

### Patient characteristics

In total, 25 patients with relapsed or refractory neuroblastoma received dinutuximab beta plus chemotherapy as part of one of the two compassionate use programs. Chemotherapy with irinotecan (50 mg/m^2^/day) plus temozolomide (100 mg/m^2^/day) (TEMIRI) on days 1–5 of each 21-day cycle was received by 24 patients and the remaining patient received topotecan (1.5 mg/m^2^/day), as they previously had PD whilst receiving TEMIRI plus bevacizumab. Patient baseline demographics, disease characteristics and the details of first-line treatment are shown in **Table 1** and **Supplementary Table 1**. The median age of the patients at diagnosis was 35.1 months (range 6.7–99.7), 11 (44%) patients had *MYCN* amplification, and 16 (64%) had unfavorable histology. The majority of patients (84%) had metastatic disease at diagnosis, 16 of whom (76%) had two or more metastatic sites, the most common of which were bone, bone marrow, lymph nodes, and liver. Most patients (72%) had received first-line treatment according to the HR-NBL SIOPEN protocol and 14 (56%) patients had received dinutuximab beta maintenance therapy in the first-line setting.

**Table 1:** Patient and disease characteristics at diagnosis and details of first-line treatment.

Category	Patients (N=25)
Age, months	
Mean	39.1 ± 20.38
Median (range)	35.1 (6.7–99.7)
Sex	
Male	13 (52)
Female	12 (48)
INSS disease stage at diagnosis	
Stage 4	21 (84)
Stage 3 with *MYCN* amplification	1 (4)
Stage 3 without *MYCN* amplification	2 (8)
Stage 2	1 (4)
*MYCN* amplification	
Amplified	11 (44)
Not amplified	12 (48)
Unknown	2 (8)
Histopathology	
Unfavorable	16 (64)
Favorable	1 (4)
Neuroblastoma (not other specified)	8 (32)
Primary tumor	
Abdomen	20 (80)
Adrenal	8 (32)
Chest	3 (12)
Pelvis	1 (4)
Neck	1 (4)
Number of metastatic compartments at diagnosis	
1	5 (20)
2	6 (24)
3	7 (29)
4	1 (4)
5	2 (8)
Metastatic sites at diagnosis^1^	
Bone	17 (68)
Bone marrow	16 (64)
Lymph nodes	11 (44)
Liver	4 (16)
Central nervous system	1 (4)
Skin	1 (4)
Lungs	1 (4)
Second tumor in abdomen	1 (4)
First-line treatment	
HR-NBL-SIOPEN	18 (72)^2^
GPOH/NB2004 protocol	1 (4)
LINES protocol	3 (12)
CHOP protocol POG 9640 (modified N7) Unknown induction	1 (4) 1 (4)
Surgery only	1 (4)
Patients who completed first-line treatment	10 (40)
Anti-GD_2_ immunotherapy as first-line maintenance	14 (56)
Dinutuximab beta only	11 (44)
Dinutuximab beta + IL-2	2 (8)
Dinutuximab beta + IL-2 and GM-CSF	1 (4)

Data are n (%) unless stated otherwise.

^1^Subgroups are not mutually exclusive, 20 (80%) of patients had more than one metastatic site; ^2^COJEC induction n=16, modified N7: n=2; ^3^subgroups are not mutually exclusive, >1 additional treatment was received following induction therapy in nine patients; ^4^One patient received irinotecan and temozolomide plus bevacizumab.

COJEC, cisplatin, carboplatin, cyclophosphamide, vincristine, etoposide; IL-2, interleukin 2; LINES protocol Group 8: etoposide, carboplatin, doxorubicin, vincristine, cyclophosphamide, radiotherapy and 13-cis retinoic acid; GM-CSF, granulocyte-macrophage colony-stimulating factor; GPOH/NB2004 protocol, two cycles of N8 (topotecan, cyclophosphamide, etoposide) – randomized, followed by six alternating courses of N5 (vindesine, cisplatin, etoposide) and N6 (vincristine, dacarbazine, ifosfamide, doxorubicin); INSS, International Neuroblastoma Staging System; MIBG, iodine-131-metaiodobenzylguanidine; modified N7 (CHOP protocol), high-dose cyclophosphamide plus doxorubicin/vincristine and cisplatin/etoposide; TEMIRI, irinotecan, temozolomide; TVD, topotecan, vincristine, doxorubicin.

Of the 25 patients who received dinutuximab beta plus chemotherapy, 20 (80%) received it for relapsed disease (1^st^ relapse n=12; 2^nd^ relapse n=6; 3^rd^ relapse n=1; 5^th^ relapse n=1), including five who had actively progressing disease at the time chemoimmunotherapy was initiated. The remaining five (20%) patients received chemoimmunotherapy for refractory disease, including two whose disease was actively progressing at the time of initiation (**Table 2**). Most patients were heavily pre-treated prior to receiving dinutuximab beta plus chemotherapy. Of the 20 patients with relapsed neuroblastoma, 8 were receiving chemoimmunotherapy to treat their second or later relapse. Twenty-one patients (84%) had received systemic treatment immediately prior to commencing chemoimmunotherapy.

**Table 2:** Disease characteristics prior to treatment with chemoimmunotherapy.

	Patients (N=25)
Reason for chemoimmunotherapy	
Refractory disease^1^	5
Primary tumor and metastases	4
Metastases only	1
Relapsed disease^2^	20
Local (primary tumor only)	2
Distant	11
Combined	7
Number of relapses at the time of chemoimmunotherapy initiation	
1	12
2	6
3	1
5	1
Timing of first relapse^3^	
During first-line treatment	10
During induction	4
During radiotherapy	1
During treatment for MRD	5^1^
At the end of first-line treatment	1
After the end of first-line treatment	9
<6 months	2
6–12 months	3
>12 months	4
Number of metastatic compartments when chemoimmunotherapy was initiated	
Primary tumor only (no metastases)	2
1	11
2	6
3	4
4	1
5	1
Metastatic sites when chemoimmunotherapy was initiated^4^	
Bone	15
Bone marrow	12
Lymph nodes	9
Liver	5
Lung	1
Soft tissue	2
Previous treatment lines included^4^	
Megachemotherapy with ASCT	18
MIBG therapy	6

Data are n.

^1^Including two patients who had progressive disease when chemoimmunotherapy was initiated;

^2^Including five patients who had progressive disease when chemoimmunotherapy was initiated;

^3^Including four patients during treatment with dinutuximab beta and one patient after the first cycle of 13-cis retinoic acid;

^4^subgroups are not mutually exclusive. ASCT, autologous stem cell transplant; MIBG, iodine-131-metaiodobenzylguanidine; MRD, minimal residual disease

The median time from diagnosis to first treatment failure was 12.7 months (range 3.2–44.0) and the median time from initial diagnosis to the initiation of chemoimmunotherapy was 18.8 months (range 4.5–98.5).

Eight patients received additional treatment following chemoimmunotherapy, the details of which are outlined in **Supplementary Table 2**.

### Tumor response

Patients received 1–10 cycles of dinutuximab beta plus chemotherapy (mean 5). A CR and a PR were each achieved in eight of 25 patients (32%), giving a best ORR of 64% (16/25) (**Table 3**). An additional five (20%) patients had SD, giving a disease control rate (DCR) of 84% (21/25). PD without any response was observed in four patients (16%). Of the 16 patients who achieved a best response of CR or PR, 11 (69%) achieved it after a maximum of 5 treatment cycles, and five (31%) after 6–8 treatment cycles (**Supplementary Table 2**). Ten of these 16 patients (63%) were still alive at data cutoff, four of whom did not receive any further treatment following chemoimmunotherapy and were alive without disease progression or relapse.

**Table 3:** Treatment response in all 25 patients receiving chemoimmunotherapy.

	Response at end of observation n (%)	Best response n (%)
Objective response rate*	11 (44)	16 (64)
Complete responses	7 (28)	8 (32)
Partial responses	4 (16)	8 (32)
Stable disease	3 (12)	5 (20)
Progressive disease	11 (44)	4 (16)
Disease control rate	14 (56)	21 (84)

*Objective response rate includes patients with complete and partial response.

Of the 14 patients who had been treated with dinutuximab beta in previous treatment lines, four achieved CR and six PR as best response (ORR 71%); two of these patients had progressed during first-line dinutuximab beta therapy. Seven (50%) of those 14 patients who had received prior dinutuximab beta were alive at data cut-off.

Of the 12 patients who were in first relapse at chemoimmunotherapy initiation, two achieved CR and three PR (ORR 42%), two had PD and five disease progression. Of the six patients treated for second relapse, four had CR, one PR (ORR 83.3%), and one progressed. The patients in third and fifth relapse achieved CR and PR, respectively. Of the 5 patients who had refractory disease at treatment, one patient achieved CR, one PR (ORR 40%) and one SD as best response; two patients demonstrated rapid PD. Six patients – two with refractory and four with relapsed disease – started chemoimmunotherapy when their disease was rapidly progression, five of whom rapidly progressed on therapy and died of disease; one patient with bone disease only achieved CR.

### Tumor response based on tumor site

Overall, the best response rates were observed in patients with bone marrow disease, and the worst response in those with large primary tumors and/or massive liver involvement.

Two patients had relapses at the primary tumor site only, both of whom developed PD during chemoimmunotherapy and died (one of them achieved CR as best response). Of the seven patients with metastatic disease that was confined to the bone, three (43%) had a CR, two (29%) had a PR (ORR 71%), and one had SD (DCR 86%); one patient developed PD and died. Two patients who had isolated neuroblastoma in the bone marrow achieved a CR, and two patients who only had metastatic disease in the liver experienced PD and died (one of them transiently achieved SD as best response). Of the two patients with soft tissue lesions, one achieved CR and one PR. Lymph node involvement was observed in nine patients, three of whom demonstrated CR, three PR, two SD and one PD. Of the 12 patients with involvement of more than one metastatic compartment, two had a CR, six had a PR (ORR 67%), and two had SD as the best response (DCR 83%); the remaining two patients (17%) had PD.

### Survival analyses

**Figure 1:**
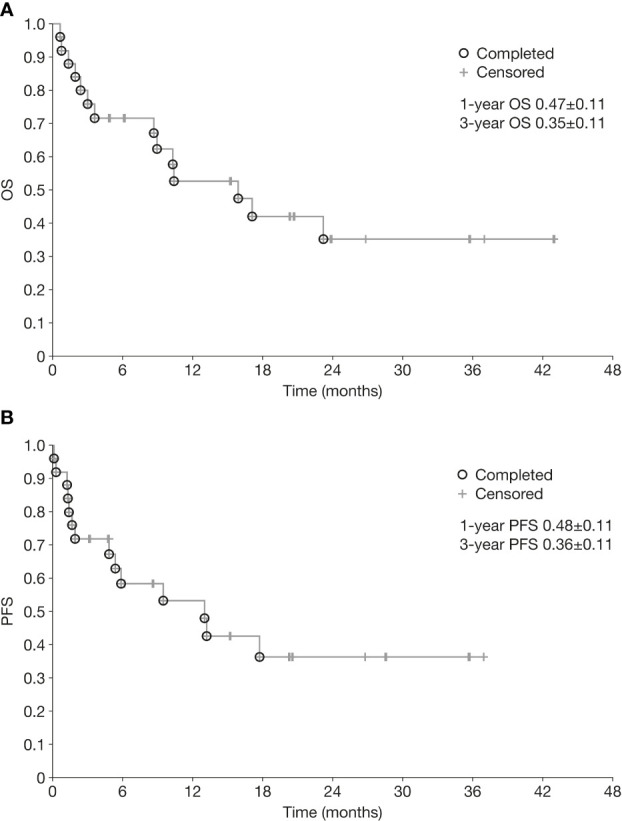
Overall survival **(A)** and progression-free survival **(B)** from initiation of chemoimmunotherapy in patients with relapsed/refractory neuroblastoma. OS, overall survival; PFS, progression-free survival.

**Figure 2:**
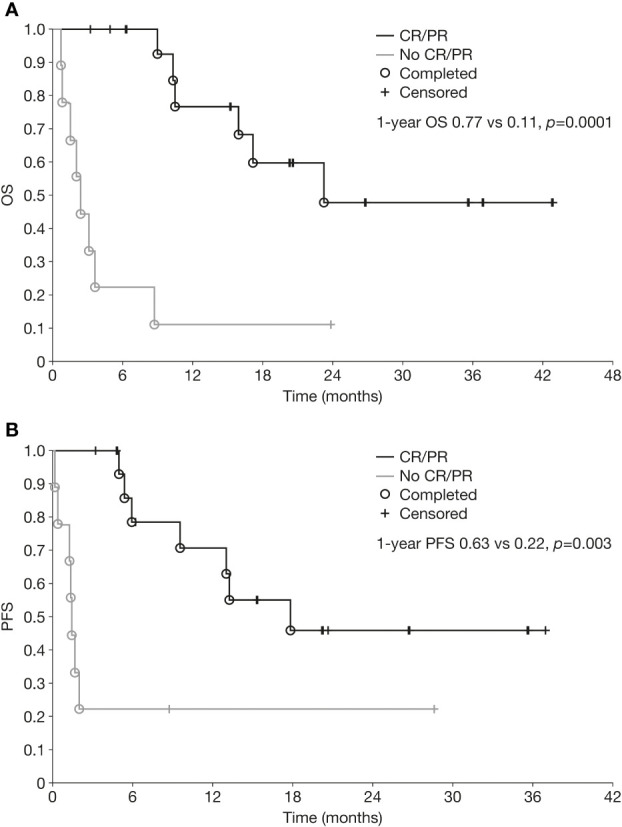
Overall survival **(A)** and progression-free survival **(B)** from initiation of chemoimmunotherapy in patients with relapsed/refractory neuroblastoma who achieved a partial or complete response versus those who did not. CR, complete response; OS, overall survival; PFS, progression-free survival; PR, partial response.

Kaplan-Meier curves for OS and PFS for the overall population are shown in **Figure 1**. Median OS and PFS from the initiation of chemoimmunotherapy were 10.3 months (range 0.7–43.0) and 6.3 months (range 0.2–37.0), respectively. The OS rate was 47% at 1 year and 35% at 3 years, and the PFS rate 48% and 36% at 1 year and 3 years, respectively. One-year OS and PFS rates were significantly better in patients who achieved CR or PR than in those who did not (1-year OS 77% vs 11%, *p*=0.0001; 1-year PFS 63% vs 22%, *p*=0.003) (**Figure 2**).

Of the 25 patients included in this analysis, 11 (44%) were alive at data cutoff, eight of whom completed therapy (with a median time from the beginning of therapy of 22.0 months [range 3.2–37.0]), and three were still receiving therapy. Of the 14 patients who died, 13 died of PD and one died of toxicity unrelated to chemoimmunotherapy almost 9 months after the end of this treatment.

### Factors affecting outcomes

In the univariate analysis of factors affecting tumor response, previous therapy with dinutuximab beta (*p*=0.03) and previous therapy with megachemotherapy plus ASCT (*p*=0.04) were the only factors with a significant effect on tumor response (**Supplementary Table 3**). Type of relapse demonstrated a borderline statistically significant effect on survival, with higher survival rates observed for patients with metastatic relapse only than for those who had a combined (primary and metastatic) relapse (*p*=0.05). All other factors analyzed were not significantly associated with survival or tumor response.

### Safety

No severe or unexpected toxicities were observed. Most patients did not require dinutuximab beta dose reductions. Dinutuximab beta was initiated at 50% of the full dose in one patient, as they had experienced toxicities with dinutuximab beta in the first-line setting; further cycles were given at the full dose. Temporary dose reductions were required in two patients: one patient received 60% of the dose in the last cycle (cycle 6) due to an infection in the central venous catheter, necessitating its removal, and the other patient received 90% of the dinutuximab beta dose in the first cycle due to large skin lesions potentially associated with dinutuximab beta. However, the latter patient received another four cycles of full dose dinutuximab beta without experiencing this complication again. No other dose reductions were necessary. However, the infusion rate of dinutuximab beta was reduced due to severe pain in one patient with bone progression and intense pain prior to treatment. The chemotherapy dose was reduced in one cycle for three patients due to myelosuppression.

The only grade 4 AEs observed were hematologic toxicities. There were four cases of grade 3 capillary leak syndrome (**Table 4**), which were managed with supportive care. Four patients had grade 3 allergic reactions, which generally presented as skin rash or bronchospasm and were manageable with standard supportive care. Diarrhea, when present, was manageable with supportive treatment. Despite standard supportive treatment before, during, and after the dinutuximab beta infusion, 10 patients had pain rated as 3–10 on a 10-point scale: three patients reported 3 points, three 4 points, one 5 points, two 8 points and one 10 points. All patients had inpatient supportive therapy for the entire duration of dinutuximab beta treatment. While patients with 2–5 points on the pain scale only needed paracetamol to reduce pain, patients with 8–10 points required morphine boluses and/or short-term dose increases of basal morphine infusion. The patient who reported 10 points on the pain scale also required short (a few hours) interruption of the dinutuximab beta infusion. However, this patient had severe pain caused by bone metastases before the start of dinutuximab beta therapy. No severe neurotoxicities were observed.

**Table 4:** Grade 3 or 4* toxicities in all 25 patients receiving chemoimmunotherapy.

	Patients, n (%)
Anemia	16 (64)
Neutropenia	18 (72)
Thrombocytopenia	12 (48)
Transaminases increase	10 (40)
Capillary leak syndrome	4 (16)
Allergic reaction	4 (16)
Diarrhea	1 (4)

*The only grade 4 events were hematologic.

Late complications of treatment were evaluated in patients who stopped taking dinutuximab beta plus chemotherapy. Hypothyroidism was reported in four patients, chronic kidney disease in one patient, and focal nodular hyperplasia of the liver in another (relapse was excluded by histopathologic examination). Patients had normal blood counts, except one with leukopenia and neutropenia in whom bone marrow examination did not confirm bone marrow dysfunction.

## Discussion

Our experience from two compassionate use programs indicates that dinutuximab beta and chemotherapy in patients with relapsed or refractory neuroblastoma is feasible and associated with encouraging tumor responses and survival rates. We observed an ORR of 64% (DCR 84%), with stable remission for up to 3 years following treatment completion. The OS rate was 47% at 1 year and 35% at 3 years. The combination therapy was well tolerated in patients who received dinutuximab beta for the first time and in those who had received it during first-line treatment. Toxicity associated with chemoimmunotherapy was manageable and rarely required dinutuximab beta dose modification or reduction.

Although initially 5 treatment cycles of dinutuximab beta plus chemotherapy were planned, our data indicate that in 20% of patients, best response, was achieved after 5 or more treatment cycles. In the event of continuous regression in the tumor size from cycle to cycle or disease stabilization, it seemed reasonable to continue treatment until no further improvement was observed for at least 2 further cycles, or until disease progression. It is currently uncertain which treatment patients should receive after they achieved a CR with chemoimmunotherapy. Similar to frontline treatment concepts, a consolidation strategy may my beneficial; however, the type of consolidation is subject to clinical research. One option is to administer consolidation therapy using hematopoietic stem cell transplantation (either auto- or haploidentical) followed by dinutuximab beta, use dinutuximab beta alone or in combination with other novel drugs (e.g. checkpoint inhibitors) or continue dinutuximab beta plus chemotherapy. We continued chemoimmunotherapy for at least 2 further cycles after a CR/best response was achieved. Treatment was stopped if no further regression was observed during two consecutive assessments, or in the event of disease progression. Our data suggest that additional cycles of immunotherapy may be beneficial for some patients and should be administered if they do not experience side effects.

As most of our patients had at least disease stabilization following chemoimmunotherapy, modification of the treatment regimen may further improve outcomes, for example, using more selective and intensive chemotherapy, increasing the dose of dinutuximab beta, or possibly adjusting the treatment schedule (e.g. shorter intervals between treatment cycles). The choice of chemotherapy may be an important factor when optimizing outcomes. As shown here, TEMIRI is feasible, effective and has low toxicity, and may be given at the same time as dinutuximab beta. To identify the optimal chemotherapy, it is necessary to evaluate the pharmacokinetics of the therapy, markers of immunologic treatment response, and the influence of leukopenia/neutropenia on treatment results. It will need to be kept in mind that more aggressive chemotherapy also induces toxicities (mainly myelotoxicities) that may cause delayed administration of the next chemoimmunotherapy cycle due to prolonged recovery times, eventually impacting on long-term survival. It is also important to optimize the treatment schedule for chemoimmunotherapy. It is uncertain whether the 3-week interval between cycles we used here is the most suitable one for all patients. Patients with rapid disease progression may benefit from shorter intervals. Conversely, in patients who have been heavily pre-treated, longer intervals between cycles may be necessary in order to decrease hematologic toxicities often observed in heavily pre-treated patients.

A clinical study (BEACON) investigating dinutuximab beta in combination with topotecan/temozolomide has recently been completed (36). First results presented at the recent American Society of Clinical Oncology (ASCO) meeting 2022 demonstrated an ORR of 35% in the group treated with chemoimmunotherapy (n=43) and 18% in the group receiving chemotherapy only (n=21; risk ratio 1.66, 80% confidence interval [CI] 0.9−3.06, *p*=0.19) (25). The 1-year PFS rates were 57% and 27% in the chemoimmunotherapy and the chemotherapy only group, respectively (hazard ratio 0.63, 95% CI 0.32−1.25, *p*=0.19) (25). Twelve patients in the chemotherapy only arm crossed over to receive dinutuximab beta at progression (25). Encouraging results with dinutuximab beta combined with chemotherapy have also recently been reported in a single-center study in Turkey that included 19 patients with relapsed/refractory neuroblastoma (37). Chemoimmunotherapy resulted in an ORR of 63%, with six patients achieving CR and six PR (37); however, follow-up was shorter in comparison to our study.

The results of our study compare also favorably with those of the initial randomized study of the combination of dinutuximab, a similar antibody to dinutuximab beta, with irinotecan and temozolomide plus GM-CSF in patients with relapsed/refractory neuroblastoma (23). In that study, the ORR with dinutuximab plus chemotherapy was 53% in the randomized cohort (23) and 41.5% in the expansion cohort (24) in patients experiencing their first relapse/refractory events, compared with an ORR of 64% in the current study, in which patients did not receive GM-CSF. Treatment outcomes as well as factors influencing response were also evaluated in a retrospective cohort of 143 patients receiving a similar treatment regimen to that reported by Mody (23) for the first or subsequent relapses (38). The ORR was 49%, with a median response duration of 15.5 months. No clear factors influencing response were identified (38). Chemotherapy with a higher dose of temozolomide compared to the Mody study (150 mg/m^2^/day in comparison to 100 mg/m^2^/day (23)) plus GM-CSF was also investigated in combination with the humanized 3F8 antibody naxitamab in heavily pretreated patients (39), which resulted in an ORR of 64%, with 37% of patients achieving CR/PR (39). As it remains unclear if the immunologic response is primarily mediated by antibody-dependent cellular cytotoxicity and natural killer (NK) cells (21, 40) or neutrophils and macrophages (8, 24), further research is required to evaluate the role of GM-CSF.

A pilot study investigated the combination of the anti-GD2 antibody hu14.18K322A, chemotherapy and parental NK cells in heavily pre-treated patients with refractory/recurrent neuroblastoma (41). The chemotherapy regimen administered was complex and consisted of cyclophosphamide/topotecan in cycles 1–2, irinotecan/temozolomide in cycles 3–4, and ICE in cycles 5–6. The toxicity profile observed in this study supports the importance of selecting appropriate chemotherapy for heavily pre-treated patients with relapsed/refractory neuroblastoma. All patients experienced myelosuppression, and the majority of patients reported pain; four patients discontinued therapy due to adverse events. Hu14.18K322A plus chemotherapy and NK cells resulted in an ORR of 62% (8/13), with four CRs, one very good PR, and three PRs. OS at 1 year was 77%, compared with 47% in our whole cohort and 77% in patients who achieved CR/PR.

Promising early response data have also been reported for chemoimmunotherapy in the first-line setting in patients with high-risk neuroblastoma (26, 42). Adding Hu14.18K322A to induction chemotherapy improved early objective responses and achieved encouraging 3-year event-free survival rates (26). Dinutuximab was also evaluated in combination with induction chemotherapy in a retrospective case series of six patients with newly-diagnosed high-risk neuroblastoma (42). Treatment was well tolerated and all patients achieved a response, including four patients with a CR (42). The use of dinutuximab and GM-CSF alongside induction chemotherapy has also recently been evaluated in a COG single-arm pilot study, with 33 of 42 patients achieving CR or PR and only two patients experiencing PD during treatment. The treatment was well-tolerated and a randomized Phase III trial is now being planned to further evaluate chemoimmunotherapy in the induction phase (27).

The limitations of our study include its retrospective nature and the heterogeneity of the patient population. As the treatment was not planned prospectively, the number of cycles and treatment following chemoimmunotherapy was dependent on clinical decisions, which might have influenced the results. In addition, our cohort has a much higher number of relapsed than refractory patients, which may have also affected response rates. Moreover, patients with actively progressing disease as well as patients with disease stabilized with other treatments were included. In future prospective studies, these patient groups should be analyzed separately.

Our findings show that combination therapy with dinutuximab beta and TEMIRI in patients with relapsed or refractory neuroblastoma is feasible and well tolerated, with encouraging response rates and survival data. No severe side effects were observed in heavily pre-treated patients, including those who had previously been treated with dinutuximab beta. This chemoimmunotherapy combination is a promising treatment option for patients with relapsed/refractory neuroblastoma and should be further explored in clinical studies.

## Data availability statement

The original contributions presented in the study are included in the article/**Supplementary Material**. Further inquiries can be directed to the corresponding author.

## Ethics statement

The compassionate use programs involving human participants were reviewed and approved for each patient individually by the Bioethical Committee of the District Medical Chamber in Krakow, Poland, or by the local committee in Greifswald, Germany. Written informed consent to participate in this study was provided by the participants’ legal guardian/next of kin.

## Author contributions

Conceptualization by AW. Formal analysis by AW and HL. Collection, compilation, and validation of clinical data by AW, AZ-T, KE, WB, KP-W and HL. Methodology by AW and HL. Supervision and validation by HL and WB. Writing – original draft preparation by AW. Writing – review and editing by HL and WB. All authors reviewed and approved the final draft of the manuscript.
