# Supplementary material for: Dinutuximab beta combined with chemotherapy in patients with relapsed or refractory neuroblastoma
Source: Front Oncol. 2023 Feb 3;13:1082771. doi: 10.3389/fonc.2023.1082771 (PMC9936065; doi:10.3389/fonc.2023.1082771)
Supplement: Supplementary file 1 [file Table_1.docx]

**Supplementary materials**

**Supplementary Table 1:** Patient and disease characteristics prior to chemoimmunotherapy for each individual patient

| Pt | Age at diagnosis | Stage at diagnosis | Metastases at diagnosis | *MYCN* status | First-line therapy | First-line therapy completed | Time from diagnosis to ChIT (months) | Status of disease at ChIT | Progression at ChIT beginning | Treatment of previous relapses | Previous anti-GD2 therapy | Sites involved at ChIT beginning |
| --- | --- | --- | --- | --- | --- | --- | --- | --- | --- | --- | --- | --- |
| 1 | 3 yrs 10 m | 3 | No | Amplified | HR-NBL-SIOPEN + 2 TVD | Yes | 26.3 | 2^nd^ relapse | No | TEMIRI, TVD, MIBG, surgery (1^st^ relapse) | Yes – DB | Primary tumor |
| 2 | 3 yrs 4 m | 4 | BM, LN | Amplified | HR-NBL-SIOPEN + 2 TVD | No | 19.8 | 1^st^ relapse during MRD treatment | No | TOTEM, MIBG | Yes – DB + IL-2 | Bones  Primary tumor |
| 3 | 7 yrs 2 m | 2 | No | Not amplified | Surgery | Yes | 91.3 | 2^nd^ relapse | No | HR-NBL-SIOPEN; TVD, TEMIRI (1^st^ relapse), TEMIRI, bevacizumab, MIBG (2^nd^ relapse) | Yes – DB | Bones, BM, LN |
| 4 | 4 yrs 1 m | 3 | No | Amplified | HR-NBL-SIOPEN + 3 TVD | No | 18.8 | 1^st^ relapse during MRD treatment | Yes | VP/Carbo, TEMIRI + bevacizumab, surgery (spinal cord decompression) | Yes – DB | Primary tumor, BM, liver |
| 5 | 1 yr 12 m | 4 | Bones, BM, LN | Amplified | HR-NBL-SIOPEN | No | 11.7 | 1^st^ relapse during MRD treatment | Yes | TEMIRI, GPOH/N6 | Yes – DB | Liver |
| 6 | 4 yrs 1 m | 4 | Liver | Amplified at relapse but not amplified at diagnosis; | HR-NBL-SIOPEN | Yes | 98.0 | 2^nd^ relapse | No | VP/Carbo, CADO, TOTEM, surgery (1^st^ relapse) | No | BM, soft tissue tumor |
| 7 | 2 yrs 3 m | 4 | Bones, BM, CNS | Unknown | HR-NBL-SIOPEN + 2 TVD | No | 29.5 | 1^st^ relapse during RTX | No | TEMIRI + bevacizumab, MIBG + BuMel, DB | Yes – DB | Bones |
| 8 | 3 yrs 5 m | 3 | No | Not amplified | LINES modified:  7 VP/Carbo,  3 CADO, TEMIRI, TVD, 2 surgeries, RTX | Yes | 56.6 | 5^th^ relapse  (3 local, 2 disseminated) | No | 9 docetaxel/ ifosfamide/cisplatin, 1 x cisplatin/ doxorubicin cyclophosphamide/ etoposide, Ifosfomide, VCR/VP+DTIC, MIBG, BuMel, 2 surgeries, RTX, DB (relapses 1–4) | Yes – DB | Primary tumor, BM, LN |
| 9 | 4 yrs 1 m | 4 | Bones, BM, LN, liver, lungs | Amplified | HR-NBL-SIOPEN | No | 7.8 | Refractory – progression after induction | Yes | TVD, TEMIRI | No | Bones, BM, LN, liver,  primary tumor |
| 10 | 2 yrs 10 m | 4 | Bones, BM, 2^nd^ tumor in abdomen | Amplified | HR-NBL-SIOPEN | No | 14.3 | 3^rd^ relapse | Yes | TVD (1^st^ and 2^nd^ relapse), TEMIRI (2^nd^ relapse), TEMIRI + bevacizumab (3^rd^ relapse), MIBG, BuMel, RTX (2^nd^ relapse) | No | Bones |
| 11 | 1 yr 10 m | 4 | Bones, BM, LN | Not amplified | HR-NBL-SIOPEN | No | 4.5 | Refractory | No | TVD | No | Bones, BM, LN, primary tumor |
| 12 | 2 yrs 1 m | 4 | Bones, BM, liver, LN | Not amplified | HR-NBL-SIOPEN + 2 TVD + 3 TEMIRI | No | 12.6 | 1^st^ relapse after 1^st^ cycle of 13-cis RA | No | TEMIRI (1 cycle) | No | Liver |
| 13 | 3 yrs 9 m | 4 | Bones, BM | Not amplified | HR-NBL-SIOPEN | No | 10.7 | 1^st^ relapse at surgery | No | TVD, BuMel, RTX (1^st^ relapse) | No | BM |
| 14 | 6 m | 4 | Bones, BM, liver, LN, skin | Not amplified | LINES | No | 18.0 | Refractory | No | TOTEM | No | Primary tumor, bones, BM, LN, liver, lungs |
| 15 | 3 yrs 10 m | 4 | Bones, LN | Not amplified | HR-NBL-SIOPEN + 2 TVD + MIBG | Yes | 19.8 | 1^st^ relapse | No | GPOH | Yes – DB | Bones |
| 16 | 8 yrs 2 m | 4 | Bones, BM, LN | Not amplified | HR-NBL-SIOPEN + 2 TVD + 2 TEMIRI+ vaccine | Yes | 35.8 | 1^st^ relapse | No | TEMIRI | Yes – DB | Bones, LN |
| 17 | 2 yrs 5 m | 4 | Bones | Not amplified | HR-NBL-SIOPEN + 2 TVD + 1 TEMIRI | No | 15.5 | Refractory with progression before RTX | Yes | 2x cisplatin/ doxorubicin/ cyclophosphamide/ etoposide, 4 X TEMIRI | No | Bones |
| 18 | 1 yr 6 m | 4 | Bones | Not amplified | LINES | No | 17.0 | 1^st^ relapse after surgery | No | TVD, TEMIRI, MIBG + BuMel | No | Bones, BM, LN, primary tumor |
| 19 | 3 yrs 1 m | 4 | BM | Amplified | HR-NBL-SIOPEN + 2 TVD | Yes | 20.2 | 1^st^ relapse | No | TEMIRI + VCR | Yes – DB | Primary tumor |
| 20 | 2 yrs 6 m | 4 | Bones, BM, LN | Amplified | HR-NBL-SIOPEN | No | 6.6 | Refractory | No | TEMIRI | No | BM |
| 21 | 2 yrs 3 m | 4 | BM, LN | Amplified | HR-NBL-SIOPEN + TVD | No | 18.7 | 1^st^ relapse – during MRD treatment | Yes | TEMIRI + bevacizumab | Yes – DB | Primary tumor, bones, BM, LN |
| 22 | 3 yrs | 4 | Bones, BM | Unknown | GPOH + TVD + TEMIRI + CE | Yes | 64.9 | 2^nd^ relapse | Yes | TEMIRI + DB (1^st^ relapse)  RIST (2^nd^ relapse) | Yes – DB | Bones |
| 23 | 4 yrs | 4 | Bones | Amplified | HR-NBL-SIOPEN + 6 TVD | Yes | 53.2 | 2^nd^ relapse | No | TEMIRI + bevacizumab, surgery, megaCT (2^nd^ procedure), RTX | Yes – DB + IL-2 | LN, soft tissue |
| 24 | 3 yrs 8 m | 4 | Bones, BM | Not amplified | POG 9640  (modified N7, 2 x MIBG, ASCT, proton therapy, immunotherapy | Yes | 58.7 | 2^nd^ relapse | Yes | GPOH (N5, N6, N8) + RTX + TEMIRI + DB (1^st^ relapse)  TOTEM + Topo/Cyclo + Crizotinib (2^nd^ relapse) | Yes – dinutuximab + IL-2 + GM-CSF | Primary tumor, bones, BM |
| 25 | 2 yrs 1 m | 4 | Bones, BM, LN | Not amplified | Chemotherapy only | No | 6.43 | 1^st^ relapse – during induction | No | TEMIRI | No | Bones |

ASCT, autologous stem cell transplantation; BM, bone marrow; BuMel, busulfan-melphalan; CADO, cyclophosphamide, doxorubicin, vincristine; Carbo, carboplatin; ChIT, chemoimmunotherapy; CNS, central nervous system; Cyclo, cyclophosphamide; DB, dinutuximab beta; GM-CSF, granulocyte-macrophage colony stimulating factor; GPOH, two cycles of N8 (topotecan, cyclophosphamide, etoposide) – randomized, followed by six alternating courses of N5 (vindesine, cisplatin, etoposide) and N6 (vincristine, dacarbacine, ifosfamide, doxorubicin); HR-NBL, high risk neuroblastoma; IL-2, interleukin-2; LINES, etoposide, carboplatin, doxorubicin, vincristine, cyclophosphamide, radiotherapy and 13-cis retinoic acid; LN, lymph node; m, month; megaCT, megachemotherapy; MIBG, iodine-131-meta-iodobenzylguanidine; MRD, minimal residual disease; N7, cyclophosphamide, doxorubicin, vincristine, cisplatin, etoposide; POG, pediatric oncology group; pt, patient; RA, retinoic acid; RIST, temozolomide, irinotecan, rapamycin, dasatinib; RTX, radiotherapy; SIOPEN, International Society of Paediatric Oncology European Neuroblastoma Group; TEMIRI, temozolomide, irinotecan; Topo, topotecan; TOTEM, temozolomide, topotecan; TVD, topotecan, vincristine, doxorubicin; VCR, vincristine; VP, etoposide; yr(s), year(s)

| **Pt** | **Number of ChIT cycles** | **Best response** | **End of treatment response** | **Treatment after ChIT** | **Time (months) from ChIT to death/last observation** | **Status at final observation (31 January 2022)/death** |
| --- | --- | --- | --- | --- | --- | --- |
| 1 | 5 | CR – Cycle 2 | PD | No | 8.9 | DOD |
| 2 | 6 | CR – Cycle 5 | CR | No | 37.0 | Alive in CR |
| 3 | 5 | CR – Cycle 3 | CR | No | 36.0 | Alive in CR |
| 4 | 2 | PD – Cycle 2 | PD | No | 0.7 | DOD |
| 5 | 1 | PD – Cycle 1 | PD | No | 0.8 | DOD |
| 6 | 6 | CR – Cycle 4 | CR | No | 17.0 | DOD |
| 7 | 10 | PR – Cycle 8 | SD | RTX of oligometastatic disease | 27.0 | Alive with 3 stable bones lesions |
| 8 | 6 | PR – Cycle 2 | PD | No | 10.3 | DOD |
| 9 | 2 | SD – Cycle 1 | PD | N6/GPOH started – stopped due to toxicity | 2.0 | DOD |
| 10 | 9 | CR – Cycle 6 | CR | No | 20.6 | Alive in CR |
| 11 | 5 | PR – Cycle 2 | PD | MIBG (planned BuMel, not given due to progression) | 10.5 | DOD |
| 12 | 3 | SD – Cycle 2 | PD | No | 2.4 | DOD |
| 13 | 6 | CR – Cycle 3 | CR | No | 20.3 | Alive in CR |
| 14 | 4 | SD – Cycle 4 | SD | Lorlatinib, MIBG, BuMel | 8.7 | Died of toxicities after the end of ChIT |
| 15 | 9 | PR – Cycle 6 | PR* | No | 15.3 | On treatment |
| 16 | 8 | PR – Cycle 8 | PR* | No | 6.3 | On treatment |
| 17 | 2 | PD – Cycle 1 | PD | No | 1.4 | DOD |
| 18 | 6 | PR – Cycle 6 | PR* | No | 4.9 | On treatment |
| 19 | 2 | SD – Cycle 1 | PD | No | 3.1 | DOD |
| 20 | 3 | CR – Cycle 2 | CR | Continue HR-NBL-SIOPEN | 3.3 | Alive in CR |
| 21 | 2 | PD – Cycle 2 | PD | No | 3.6 | DOD |
| 22 | 2 | CR – Cycle 2 | CR | MIBG, haploSCT, DB | 23.3 | DOD |
| 23 | 7 | PR – Cycle 4 | PD | Nivolumab + DB | 43.0 | Alive in CR |
| 24 | 9 | PR – Cycle 3 | PR | No | 15.9 | DOD |
| 25 | 3 | SD – Cycle 3 | SD | MIBG + megaCT, surgery, nivolumab + DB | 53.9 | Alive with disease |

**Supplementary Table 2:** Details of chemoimmunotherapy, response data and follow-up for each individual patient

*Response at end of observation

BuMel, busulfan-melphalan; ChIT, chemoimmunotherapy; CR, complete response; DB, dinutuximab beta; DOD, died of disease; GPOH, two cycles of N8 (topotecan, cyclophosphamide, etoposide) – randomized, followed by six alternating courses of N5 (vindesine, cisplatin, etoposide) and N6 (vincristine, dacarbazine, ifosfamide, doxorubicin); haploSCT, haploidentical stem cell transplantation; HR-NBL, high-risk neuroblastoma; megaCT, megachemotherapy; MIBG, iodine-131-meta-iodobenzylguanidine; PD, progressive disease; PR, partial response; pt, patient; RTX, radiotherapy; SD, stable disease; SIOPEN, International Society of Paediatric Oncology European Neuroblastoma Group

**Supplementary Table 3:** Univariate analysis of factors associated with tumor response and survival

| **Factor analyzed** |  | **OR** | **95% CI** | ***p*-value** |
| --- | --- | --- | --- | --- |
| Age <18 months | Response | 0.55 | 0.04–7.03 | 0.65 |
|  | Survival | 0.6 | 0.05–7.6 | 0.69 |
| *MYCN* amplification | Response | 1.33 | 0.26–6.8 | 0.73 |
|  | Survival | 2.04 | 0.39–10.55 | 0.39 |
| Time of relapse – during vs after therapy | Response | 6.0 | 0.93–38.6 | 0.06 |
|  | Survival | 0.76 | 0.15–3.8 | 0.74 |
| Type of relapse – metastatic vs combined | Response | 0.2 | 0.03–1.42 | 0.1 |
|  | Survival | 0.1 | 0.01–1.04 | 0.05 |
| Previous dinutuximab beta therapy | Response | 0.12 | 0.02–0.81 | 0.03 |
|  | Survival | 0.46 | 0.09–2.32 | 0.35 |
| Previous megachemotherapy plus ASCT | Response | 0.08 | 0.007–0.9 | 0.04 |
|  | Survival | 0.81 | 0.11–5.9 | 0.84 |

ASCT, autologous stem cell transplantation; CI, confidence interval; OR, odds ratio
